# A Systematic Review of Access to General Healthcare Services for People with Disabilities in Low and Middle Income Countries

**DOI:** 10.3390/ijerph15091879

**Published:** 2018-08-30

**Authors:** Tess Bright, Hannah Kuper

**Affiliations:** International Centre for Evidence in Disability, London School of Hygiene and Tropical Medicine, London WC1E 7HT, UK; hannah.kuper@lshtm.ac.uk

**Keywords:** access, health care, low and middle income country, LMIC, universal health coverage, people with disabilities

## Abstract

*Background*: A systematic review was undertaken to explore access to general healthcare services for people with disabilities in low and middle-income countries (LMICs). *Methods*: Six electronic databases were searched in February 2017. Studies comparing access to general healthcare services by people with disabilities to those without disabilities from LMICs were included. Eligible measures of healthcare access included: utilisation, coverage, adherence, expenditure, and quality. Studies measuring disability using self-reported or clinical assessments were eligible. Title, abstract and full-text screening and data extraction was undertaken by the two authors. *Results*: Searches returned 13,048 studies, of which 50 studies were eligible. Studies were predominantly conducted in sub-Saharan Africa (30%), Latin America (24%), and East Asia/Pacific (12%). 74% of studies used cross-sectional designs and the remaining used case-control designs. There was evidence that utilisation of healthcare services was higher for people with disabilities, and healthcare expenditure was higher. There were less consistent differences between people with and without disabilities in other access measures. However, the wide variation in type and measurement of disability, and access outcomes, made comparisons across studies difficult. *Conclusions*: Developing common metrics for measuring disability and healthcare access will improve the availability of high quality, comparable data, so that healthcare access for people with disabilities can be monitored and improved.

## 1. Introduction

The WHO estimates that one billion people globally have disabilities, equating to 15% of the worldwide population [1]. There is extensive evidence that people with disabilities are on average poorer, face widespread stigma, and often face a range of exclusions, including from employment, education, and access to services [2]. It is widely believed that people with disabilities also face exclusion from healthcare services, although to date this issue has received little research attention. 

The relationship between disability and health is complex, as they are interlinked and over-lapping. A range of definitions of disability are used, but the most prevailing is that of the United Conventions on the Rights of Persons with Disabilities (UNCRPD) which states that “Persons with disabilities include those who have long-term physical, mental, intellectual or sensory impairments which in interaction with various barriers may hinder their full and effective participation in society on an equal basis with others” [3]. By definition, therefore, people with disabilities experience an impairment related to a health condition, for instance they may have visual impairment because of diabetes. 

The link between disability and poor health can also arise through other pathways. For instance, an impairment (e.g., physical impairment) may lead to further health issues (e.g., bed sores due to low mobility). People with disabilities often occupy a marginalised position in society, and so may be more vulnerable to poor health due to poverty and adverse living conditions (e.g., vulnerability to injuries) [2]. This means that on average people with disabilities will have poorer health than people without disabilities [1]. As an example, a study across 30 countries found that children with disabilities were more likely to report having a serious health problem in the last 12 months compared to their peers without disabilities [4]. Overall, therefore, people with disabilities may on average have a greater need for healthcare services, both because of their impairment and their vulnerability to poor general health. 

People with disabilities may also face challenges in accessing healthcare services, despite their greater need, which can contribute to poorer health. Services and/or transport may be physically inaccessible to people with certain impairments. People with disabilities may experience stigma and discrimination at the point of care, which can discourage them from attending. The skills and experience of healthcare professionals may be inadequate to provide a quality service (e.g., difficulties communicating with people with hearing or intellectual impairment). The cost of seeking services may be prohibitive to people with disabilities, both on account of on average higher levels of poverty as well as the additional costs incurred when seeking care (e.g., need for accessible transport or for a carer to attend). As a result of these different barriers, people with disabilities may have poorer access to healthcare services, despite their higher need. 

Failure to address access of people with disabilities to healthcare services may have profound implications. The UNCRPD specifically states that people with disabilities must have access to general and specialist healthcare (articles 25 and 26), and so exclusion from services will be an infringement on human rights since this convention has been ratified by more than 170 countries [3]. Furthermore, the achievement of Universal Health Coverage (UHC) is a key target within the Sustainable Development Goals. UHC means ensuring access to health services for all by expanding the breadth of the population covered, with the depth of services that they need, without suffering financial hardship. This target would not be met without a specific focus on people with disabilities, since they make up a large group and may be excluded from both general and specialist services. 

Despite the importance of this topic, access to healthcare among people with disabilities has received little attention in research. One challenge is the lack of consensus in how access is measured. Monitoring frameworks for UHC often recommend measuring utilization of services, yet this may be an inadequate measure of coverage or met needs for healthcare, since people with disabilities may have higher healthcare needs [5,6]. Furthermore, assessing uptake alone is insufficient; attention is also needed on the quality and affordability of services, as acknowledged within the UHC framework [7].

Access to healthcare among people with disabilities is an important issue that has not been reviewed systematically to date. Assessing access to health is complex, and needs a considered and nuanced assessment, particularly with respect to people with disabilities. The objective of this study is to systematically review the evidence on access to general healthcare services among people with disabilities in low and middle-income countries (LMIC). Access to specialist services, including rehabilitation, will be the focus of a separate review.

## 2. Materials and Methods 

The systematic review was performed and reported according to the Preferred Reporting Items for Systematic Reviews and Meta-Analysis (PRISMA) statement [8]. The search was conducted in February 2017 to identify peer-reviewed articles that presented research findings on access to general healthcare services for people with disabilities in LMIC settings. 

### 2.1. Eligibility Criteria

Studies were eligible if they met the following criteria: (1) quantitative research that included people with disabilities and a comparison group of people without disabilities; (2) results reported access to general healthcare services for people with disabilities, in comparison to people without disabilities; and (3) research was undertaken in a LMIC as defined by the World Bank country classification 2017 [9]. Studies were excluded if the full text was not available. If multiple reports from the same study were identified, the results were either combined if they reported different results or secondary papers were excluded if the results were the same as the primary report. 

#### 2.1.1. Types of Access Measures

We defined access to health according to the definition of UHC: “all people and communities can use the promotive, preventive, curative, rehabilitative and palliative health services they need, of sufficient quality to be effective, while also ensuring that the use of these services does not expose the user to financial hardship” [7]. Thus possible outcome measures were broad and included utilisation of general health services (e.g., hospitalisation, health centre visits), coverage of preventive (e.g., vaccination), and curative services (e.g., use of tuberculosis treatment services), and adherence to medications. Access to rehabilitative services is the subject of a separate review. We also included studies that measured expenditure on health services and insurance coverage to address the issue of access without exposure to financial hardship. Further, in addition to these primary outcomes, we included data about the quality of care received and reported barriers to access as secondary outcomes (if these were measured quantitatively). We did not consider health outcomes or risk behaviours such as anthropometric measures or smoking status as measures of access to health. 

#### 2.1.2. Types of Disability Measures

Defining disability is complex and approaches have evolved over time. Early models described disability as a purely medical issue, whereby an individual has an impairment in body function or structure (e.g., hearing impairment). More recent models take a broader approach and include societal barriers that prevent people with disabilities from full participation [10]. Currently, the most widely used framework is the International Classification of Functioning, Disability, and Health (ICF) developed by the World Health Organisation [11]. This model takes in to account the impairment, activity limitations, and participation restrictions as well as considering environmental factors creating the disability. This framework is considered as a bio-psycho-social model of disability. This framework also aligns with the UNCRPD definition of disability. Studies were eligible for inclusion if they defined disability based within the ICF framework (e.g., functioning, activity limitations, or participation restrictions) or medical model definitions (i.e., specific impairments or disorders).

### 2.2. Information Sources

Six databases (EMBASE, Global Health, CINAHL, Web of Science, MEDLINE, and PSYCINFO) were searched. No limits were placed on language or date of publication. The search strategy included key words for the following three concepts: LMICs, people with disabilities, and access to health services. Terms were developed using MeSH (Medical Subject Headings used by the National Library of Medicine to index articles) or equivalent as well as from other reviews on similar topics. Boolean, truncation, and proximity operators were used to construct and combine searches for the key concepts as required for individual databases. An example of the search strategy is provided as Supplementary File S1. We also included eligible studies known to the authors.

### 2.3. Study Selection 

All studies identified through the search process were exported to a bibliographic database (EndNote version X7, Clarivate Analytics, Philadelphia, PA, USA) for removal of duplications and screening. Two review authors (Tess Bright and Hannah Kuper) independently examined the titles, and abstracts of electronic records according to the eligibility criteria. Results of the initial screening were compared and full-text records obtained for all potentially eligible studies. Two review authors (Tess Bright and Hannah Kuper) screened the full texts using eligibility criteria for final inclusion in the systematic review. Systematic reviews identified through the search were reviewed for eligible studies. If study protocols were identified, a search was made to determine whether the results of the study had been published. Any disagreements in the selection of the full text for inclusion were resolved through discussion.

### 2.4. Data Extraction and Analysis

Data were extracted into a Microsoft Excel database developed for the purposes of this review. The first author (Tess Bright) extracted all data and this was independently checked by the second reviewer (Hannah Kuper) to ensure accuracy. Any differences between the reviewers were discussed and resolved. Data were extracted on the following study components:

- General study information, including author, year of publication
- Study design, sampling, and recruitment methods
- Study setting and dates conducted
- Population characteristics including age, sex, sample size, proportion of participants with a disability
- Study measures: Means of assessing disability, means of assessing access
- Research outcomes (main findings related to access to health): where possible odds or prevalence ratios were extracted as a measure of association. In the absence of these effect estimates, *p*-values comparing the measures in people with and without disabilities were extracted

We did not conduct a meta-analysis due to the wide variation in included study designs, intervention types and outcomes measured. Instead, a narrative synthesis was undertaken. Results were summarised by outcome type (i.e., measure of healthcare access). Each outcome type was classified in terms of whether access to healthcare of people with disabilities in comparison to people without disabilities was “higher”, “lower” or not different “null”. If studies measured multiple outcomes within the same type (e.g., utilisation of hospital and health centre), studies were classified as “higher” or “lower” if at least one outcome was statistically significant and the others were null. Studies were classified as “mixed” if results for a study were higher in one variable of interest or disability domain and lower in another.

### 2.5. Risk of Bias in Individual Studies

Quality assessments of all eligible studies were carried out independently by two reviewers (Tess Bright and Hannah Kuper). We evaluated studies based on a set of criteria according to the SIGN50 (Scottish Intercollegiate Guidelines Network) checklists [12]. The criteria are outline in Table 1. 

## 3. Results

**Figure 1:**
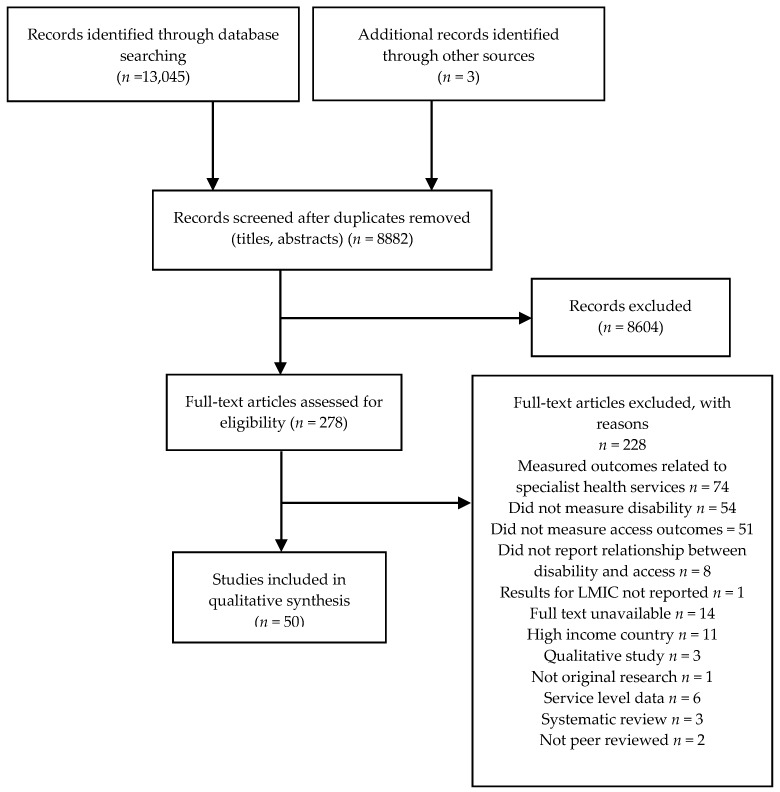
Flow chart of search results.

A total of 13,045 studies were initially identified by the electronic searches, of which 50 studies were selected for inclusion in the review. The screening process is detailed in Figure 1.

### 3.1. Study Characteristics

Table 2 summarises the characteristics of the included studies. The vast majority of included studies were conducted in 2010 or later (82%). Nearly one third (30%) of studies were conducted in sub-Saharan Africa, 24% in Latin America, 12% in East Asia/Pacific, 4% in the Middle East, 12% in South Asia, 2% in Europe and the remaining 16% were conducted across multiple regions. By country income group (World Bank Classifications), 16% were conducted in low-income countries, 34% in lower-middle, 34% in upper-middle, and 16% in countries across multiple income groups. Approximately half of studies were conducted in both urban and rural areas (48%), with a further 30% conducted in urban areas alone, and 8% in rural areas alone. For 14% of studies the location was unclear. The majority of studies were cross-sectional surveys (74%) with the remaining studies using case-control designs (26%). Of the cross-sectional studies, 80% were population-based whilst the remaining recruited participants from clinics (three studies), registries (three studies), or schools (two studies). Supplementary File S2 provides a detailed summary of included studies.

### 3.2. Participants

The studies selected for inclusion provided data for 1,510,959 people across 75 countries (not including the data from the World Health Surveys, included in the World Report on Disability). On average, people with disabilities made up 51% of the study participants in included studies. The studies included a broad distribution of people of different ages: 20% of studies included people of all ages, 32% included adults only (typically >18 years), 22% included older adults (>40 years), and 20% included children (<18 years). Two studies did not present the age of the participants. 

In terms of domains of disability, seven categories were identified: hearing impairment, visual impairment, physical impairment, mental impairment, functional difficulties, participation, and others. 48% of studies measured functional difficulties across domains of hearing, vision, walking, self-care, communicating and remembering or concentrating. Among studies that focused on impairment types, the most common category was mental impairment (includes poor mental health, intellectual, and cognitive impairment-measured in 48% of studies), followed by physical impairment (30%), hearing impairment (30%) and visual impairment (22%). Disability was measured in terms of whether people needed assistance with activities of daily living (basic or instrumental) in 6% of studies. Supplementary File S2 provides details of the disability domains and how they were measured in each study. A wide range of tools were utilized to measure disability–both self-report and clinical tools. Access outcomes were disaggregated by disability type or functional domain in 22 studies. Overall, 8 studies provided access outcome results for people with hearing impairment, 14 for mental impairment or cognitive difficulties, eight for physical impairment, and four for visual impairment.

### 3.3. Outcome Types 

The following seven outcome types were identified with respect to access to healthcare services: utilisation of services (20 studies), coverage of services (22 studies), insurance coverage (5 studies), cost/expenditure on services (8 studies), adherence to treatment (3 studies), quality of service delivery (3 studies) and barriers to access (8 studies).

### 3.4. Risk of Bias within Studies

Of the included studies, 54% were judged to have low risk of bias, 34% medium, and 12% high risk of bias. Low response rate (<70%) or insufficient information to make judgement was a concern in 26 studies. Some important aspects of a robust analysis were lacking from several studies, such as lack of reporting of confidence intervals (13 studies), or lack of adjustment for confounders (20 studies). In three studies, the methods for assessing disability were unclear. One study used a single binary question to determine whether an individual had a disability or not and one study used binary responses across several domains (e.g., seeing, hearing). These types of questions are likely to underestimate the prevalence of disability by missing milder forms of disability. Supplementary File S3 provides details of the risk of bias by domain.

### 3.5. Description of Findings Related to Healthcare Access

**Figure 2:**
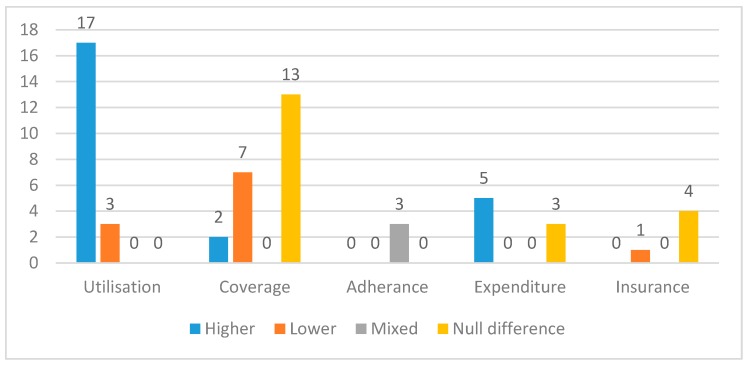
Results by outcome type.

Figure 2 provides a summary of the results from the 50 included studies, grouped by outcome type. Of the 20 studies measuring utilisation of healthcare, 17 (85%) found that utilisation of healthcare services was higher for people with disabilities compared to those without, and the remaining three studies (15%) showed lower utilisation. Twenty two studies measured healthcare coverage. The majority of studies found no difference in coverage between people with disabilities in comparison to people without (*n* = 13; 59%). Two studies found higher coverage (9%), and seven found lower coverage (32%). Three studies measured adherence, all finding lower adherence amongst people with disabilities. Eight studies measured outcomes related to health expenditure; Five studies (63%) found higher expenditure, and three studies (37%) found no difference. Of the five studies measuring insurance coverage, four (80%) found no difference, and one (20%) found lower coverage among people with disabilities. The results are discussed in more detail in the following sections. 

#### 3.5.1. Utilisation

Utilisation of healthcare services was measured using a range of outcomes in two main groups (use of primary or secondary health services, and use of tertiary (hospital) services), and by different time-periods of assessment. The outcomes included the following:

- Primary or secondary health services (13 studies)
  ○ Number of visits to health centre or public health facility in past 12 months (4 studies); 6 months (1 study); and 1–3 months (4 studies)
  ○ Access to services in past 6 months (1 study)
  ○ Home visits by a doctor in past 12 months (1 study)
  ○ Length of time since last consultation (1 study)
  ○ Clinic attendance (unspecified time frame; 1 study)
- Tertiary services (hospital) (12 studies)
  ○ Hospitalisations (inpatient admission) in the past 5 years (1 study); 12 months (7 studies), 6 months (1 study); 3 months (1 study); or over an unspecified time frame (1 study)
  ○ Hospital outpatient visit in the previous 12 months (1 study), 6 months (1 study) or one month (2 studies)

##### Utilisation: Primary or Secondary Service Use

Thirteen studies assessed utilisation of primary or secondary healthcare services, comparing people with and without disabilities (summarised in Table 3). Three different periods of recall were assessed across studies: past 12 months, 2–3 months, and one month. Five of the 13 studies measuring this outcome were conducted in Brazil. 

For utilization of services in the past 12 months, one of four studies found no significant difference between people with and without disabilities, and for the remaining study the results showed higher utilisation for people with disabilities. For utilisation in the past six months, two studies found higher utilisation and one found lower utilisation for people with disabilities. All studies found higher utilisation in the past 1–3 months amongst people with disabilities compared to those without. Regular clinic attendance over an unspecified period was lower for people with disabilities in one study. 

Finally, one study measured the length of time since last consultation and found a higher proportion of people with disabilities had sought care in the past 30 days, and a lower proportion reported they had sought care between 1–2 years ago in comparison to people without disabilities. 

Seven studies measuring utilisation of primary health services disaggregated results by domain of disability or only measured one disability domain; five by mental impairment, one hearing impairment and two physical impairment. Considering studies measuring mental impairment, in Brazil, Fujii et al. found a greater number of health care visits for adults with mental health conditions [20]. Albanese et al. reported higher community health service use for older adults (>65 years) with depression across nine LMIC [17]. Similarly, Andrade et al. found higher utilisation of general health services for adults with mental disorders in Brazil [24]. In contrast, Blay et al. found no difference in health care utilisation Brazil for people with or without mental health conditions [27]. Further, in China, Liu et al. found no difference in health service utilisation for older adults with dementia compared to those without [22].

Considering utilisation results by other disability types, Groce et al. found adults with hearing impairment had lower regular clinic attendance compared to adults without impairment in Swaziland [25]. Albanese et al. reported that community health service use was higher for older people (>65 years) with physical impairments and mobility restrictions (pooled-estimate across multiple LMICs) compared to those without [17]

##### Utilisation: Hospitalisation

Twelve studies measured hospitalisation (inpatient or outpatient) over varying periods of time (Table 4). Nearly half of these studies were conducted in Brazil (five studies). In general, occurrence of inpatient hospital admission was significantly higher amongst people with disabilities compared to people without disabilities. Outpatient visits were measured in four studies, and the majority (*n* = 3; 75%) found no significant difference between frequency of visits for people with and without disabilities, and one found a higher number of visits among those with disabilities. 

The World Report on Disability summarises results from 50 World Health Surveys and reports individuals’ care seeking behaviour by country income level [1]. The analysis found that in low-income countries, a significantly higher proportion of both males and females with disabilities (>18 years) sought inpatient and outpatient care. Higher levels of care seeking for people with disabilities was seen for people in all age groups except for those aged 60 years and older. 

Several studies disaggregated results by disability domain, but the numbers of studies in each group were too small to detect consistent patterns. Castro et al. found no significant difference in hospitalisation for people of all ages with and without hearing impairment (Brazil) [28], while Freire et al. found a higher proportion of adults with hearing impairment had been hospitalised in the past year (Brazil) [21]. Blay et al. found no difference in outpatient visits, but higher hospitalisations in the past 12 months for older adults with physical impairments (>60 years) in Brazil [27]. Similarly, both Liu et al. and Castro et al. found higher hospitalisation for adults with physical impairments in both China and Brazil respectively [22,28]. Twomey et al. reported that hospital admission did not differ between those with and without depression [23]. However, for those with functional difficulties an increase in hospitalisation in the past 3 months was found. 

#### 3.5.2. Coverage 

Table 5 summarises the results of the 22 studies measuring coverage. Coverage outcomes included:

- Care seeking when ill (over varying time periods) (9 studies)
- Coverage of specific services: HIV related (four studies); vaccination coverage (9 studies); dental visits (4 studies); maternal health outcomes (4 studies); receipt of vitamin A (1 study); and others (1 study).

##### Care Seeking When Ill

Of the nine studies measuring care seeking when ill, four found lower coverage for people with disabilities and five studies found no difference (Table 5). By disability domain, care seeking when ill was found to be lower in one study by Wanera et al. amongst adults (>50 years) in Uganda for either physical impairments, visual impairments or mental impairment, however no difference was seen for people with hearing impairment [35]. Emerson et al. found across 25 LMIC that children with intellectual disability had reduced odds of seeking care for acute respiratory infection, reduced odds of pre-packaged oral rehydration treatment for diarrhoea, and no significant difference for help seeking for fever [36]. 

##### Coverage of Specific Services

Four studies measured coverage of dental services, all of which focussed on children with intellectual disabilities Khatib et al. and Al Habeshneh et al. found lower previous dental attendance for children with intellectual disability in Egypt, and Jordan respectively [37,38,39]. Oredugba et al. and Rahim et al. found no difference in any dental treatment received amongst children with intellectual disability in Nigeria and Malaysia respectively [40].

Four studies measured indicators of maternal health coverage. None of these studies found a difference between women with and without disabilities. Results were not disaggregated by disability domain for any of the studies. 

Five studies measuring vaccination coverage for people with and without disabilities were conducted in Brazil (>60 years), India (children), Kenya (children), Peru (all ages), and Sierra Leone (adults only). In Sierra Leone, level of immunisation was lower for people with severe functional difficulties than people without. No differences were observed in other studies. Sato et al. (2015) found no significant difference in influenza vaccination coverage for people with either depression or “being bedridden”. No other studies disaggregated results by disability domain [41]. 

Higher coverage of HIV services (e.g., HIV testing) was found in two of four studies, with the remaining finding no difference. By disability domain, Abimanyi-Ochom et al. found people (aged 15–54 years) with hearing impairment had fewer months since their last HIV test (Uganda) [42]. In contrast, Bisol et al. found a no difference between people with and without hearing impairment aged 15–21 years in testing for HIV (Brazil) [43]. 

Two studies measured other indicators of coverage of health services. Freire et al. (2009) found no significant difference in time since last prostate examination in Brazilian men with and without hearing impairment, however women with hearing impairment had a significantly longer time since last Pap smears [21]. Gottleib et al. assessed vitamin A supplementation in children aged 2–4 years by disability status across 18 countries, and found lower coverage among children with disabilities in Bangladesh, Belize, Ghana, Iraq and Mauritiana [44]. No difference was found in the remaining 13 countries included in the study.

#### 3.5.3. Adherence 

Three studies measured adherence to treatment by disability status—either to HIV treatment, fluid or diet restrictions for end stage renal disease, or medication (Table 6). Of these studies, two found mixed results by impairment type, and one found adherence was lower among people without disabilities than in people without disabilities. Hannass-Hancock et al. (2015) found no difference in HIV treatment adherence in South Africa for people with cognitive difficulties compared to those without, however adherence was found to be lower for adults with mobility impairments [45]. Tavares et al. (2013) found lower adherence for people reporting difficulties in participation (Instrumental Activities of Daily Living incapacity) in Brazil (>60 years), however no difference was seen for people with cognitive impairment or depression [46].

#### 3.5.4. Health Expenditure

Table 7 shows the results of the eight studies measuring health expenditure. Expenditure outcomes included catastrophic health expenditure (three studies); total out of pocket expenditure (four studies); total medical expenses (one study) and health expenditure to income ratio (one study). Five studies (63%) found higher expenditure incurred by people with disabilities, three studies (37%) found no difference. 

Two studies reported results of expenditure amongst those with and without hearing impairment. Brinda et al. found out of pocket expenditure was higher for adults with either hearing or vision impairment in India when seeking healthcare [57]. In contrast, Brinda et al. found no difference in expenditure for adults with hearing impairment compared to those without hearing impairment in Tanzania [58].

Three studies reported expenditure for those with varying mental impairments. Brinda et al. found higher out of pocket expenditure for older adults with dementia in India [57]. Also in India, Brinda et al. found higher catastrophic expenditure for older adults with depression [59]. In contrast, Brinda et al. found no significant difference in out of pocket expenditure for adults with psychiatric morbidity in Tanzania [58].

Brinda et al. also found no difference in out of pocket expenditure for adults with physical impairment (“limb defects”) in Tanzania [58]. Finally, both Brinda et al. and Brinda et al. found no significant difference in out of pocket expenditure for adults with visual impairment compared to those without in India and Tanzania respectively [57,59].

#### 3.5.5. Health Insurance Coverage

Table 7 shows the results of the five studies measuring insurance coverage. Access to health insurance was measured in two main ways—medical payment method (out of pocket, or by insurance plan) or coverage (four studies). One study found lower coverage amongst people with disabilities, and four studies found no difference in coverage. 

#### 3.5.6. Barriers to Access

In addition to other access outcomes, eight studies measured barriers to accessing general healthcare services as a secondary outcome. The most commonly reported barriers across studies were transport difficulties, financial difficulties and attitudes of staff. People with disabilities reported experiencing greater barriers to accessing health services than people without disabilities in all five studies that made this comparison (three studies only reported barriers for people with disabilities). These results are shown in Table 8.

#### 3.5.7. Quality of Services

Three studies measured quality-related outcomes with respect to health access including: ease of access, satisfaction, and overall accessibility. Trani et al. found no difference in satisfaction with public health facilities in Afghanistan for people aged >5 years with and without disabilities [14]. In Thailand, a study by Wongkongdech et al. found that 66% of people with physical impairment of all ages ranked their accessibility to health services at a moderate level (i.e., neither high nor low), taking in to account adequacy of health personnel, respect for rights and dignity, transport, service related aspects, personal factors and costs [62]. Finally, Badu et al. found that 71% of people faced discrimination from health care providers [61].

## 4. Discussion

### 4.1. Overview of Results 

This review provides a comprehensive overview of published studies that have assessed the relationship between disability and access to general healthcare services in LMIC. Seven main groups of healthcare access outcomes were identified: health care utilisation, coverage, adherence, health insurance coverage, health expenditure, barriers and quality related outcomes. 

Utilisation is expected to be higher for some people with disabilities because by definition, people with disabilities have an underlying health condition that causes an impairment [11]. Our found some evidence to support this expectation. This review found evidence that in general both hospitalisation and utilisation of primary health services was higher for people with disabilities compared to people without disabilities. However, half of these studies were judged to have a high or moderate risk of bias, which may influence the findings. 

It is commonly believed that coverage of health services is lower for people with disabilities than people without, our review found limited evidence to support this assumption [63]. Overall, coverage outcomes were varied in this review, even within sub-groups. We found some evidence that coverage of dental services for children with intellectual impairments was lower than children without disabilities. No difference in coverage of maternal health services was seen between people with and without disabilities. Results of studies measuring coverage of HIV services, care seeking when ill, and vaccinations were varied with both higher and lower coverage reported for people with disabilities. Again these results should be interpreted in light of the poor quality of some of the included studies. 

Few studies measured outcomes related to adherence. However, results suggested a trend towards lower adherence among people with disabilities. The majority of studies that measured health insurance found no significant difference between people with and without disabilities. Results from studies measuring health expenditure showed a trend toward higher expenditure for people with disabilities. Of eight studies, five found higher expenditure for people with disabilities compared to people without. A further three studies found null difference. 

Commonly reported barriers included those related to geographic accessibility, financial accessibility and acceptability of health services. Quality of services received by people with disabilities was measured in very few studies. 

The review highlighted that a diverse range of indicators are used to measure access to health services, making it difficult to compare studies and draw strong conclusions. This highlights the need for defined, consistent metrics for measuring access to allow comparability and monitoring of progress towards UHC. Nearly half of the studies in this review were judged to have a high or moderate risk of bias, which highlights the urgent need for high quality studies to be carried out. No consistent patterns were seen by age, locality or disability domain, although the number of studies disaggregating data by these variables was small. These indicators, alongside other measures of equity such as socioeconomic status or gender are crucial for understanding progress towards UHC.

### 4.2. Consistency with Previous Literature

Few systematic reviews have been conducted on this topic to allow for comparisons to be made, but findings from high-income countries more clearly show poor healthcare access among people with disabilities. A review by Gibson et al. found that people with disabilities had restricted access to and report less satisfaction in their medical care [64]. This paper had a bias towards high-income contexts, and was based mostly on qualitative studies. In another review by Alborz et al., evidence suggested that people with learning disabilities may access general practices and dental surgeries less frequently than the general population [65]. This concurs with our findings. Alborz et al. also identified that people with learning disabilities were less likely to receive preventive healthcare. Again, this review had a focus on high-income contexts which means the results are not directly comparable to our review. Other studies from high income settings also show poorer access to healthcare among people with disabilities, including in Chile [66], and the United Kingdom, with long waiting lists and transportation being the main reported barriers in the UK [67]. The results for our review were more mixed. Often, overall coverage was very high for some indicators (e.g., for vaccination), making it difficult to find differences between groups.

Our review found that health expenditure was typically higher for people with disabilities. There is a growing body of evidence to support the link between disability and poverty [2]. Recent research in Vietnam and Nepal on social protection for people with disabilities found that spending on health care was one of the main courses of additional costs for people with disabilities that could contribute to poverty [68,69]. This aligns with the findings of our review, however further evidence is needed. 

Results from high-income contexts including the United States and Korea suggest that adherence to treatment tends to be lower for people with disabilities. A study by Park et al. found people with disabilities had lower adherence to antihypertensive medications than people without disabilities [70]. In the USA, lower adherence to prescription medication was found for post-myocardial infarction patients with disabilities [71]. Although our review identified few studies measuring adherence, the findings concur with this research from high income contexts. 

This review has highlighted that further research is needed to understand how people with disabilities are accessing health services, not just in terms of utilisation, but also coverage of preventive services, affordability of health services, and the quality of care received. In particular, there is a need to define a broader range of metrics to measure access more holistically (beyond utilization alone) and allow greater comparability of outcomes across countries. There is also a need for consistent definitions of disability to be used, in order to allow comparability across studies. 

UHC strives to achieve health for all, leaving nobody behind, and without more inclusive indicators, we will not be able to monitor progress towards this target. Although the results were varied, this review found supporting evidence that people with disabilities are being left behind on the path towards UHC. Consequently, efforts are needed to remove barriers so that access to healthcare services is made equitable for people with disabilities. The right to healthcare and rehabilitation for people with disabilities is enshrined within the UNCRPD, and within the laws and policies of most countries [3]. More efforts are therefore needed to make changes at the levels of services and programmes, rather than at the policy level. Yet currently evidence on the effectiveness of interventions that work towards achieving these changes is limited [72,73]. 

Some examples of good practice for achieving improvements in the dimensions of UHC exist from LMIC. Considering financial coverage, in Vietnam, some people with disabilities who are recipients of a Disability Allowance also receive free health insurance, which may help achieve financial protection [68]. As another example, in India a 3-year programme between 2009–2011 “Inclusion for All” was initiated by World Vision to increase awareness of HIV/AIDS amongst people with disabilities and resulted in a positive change in attitude towards people with disability in the community [74]. 

### 4.3. Strengths and Limitations of the Review 

This review has several limitations that need to be taken in to account when considering the results. The definition of access we adopted may not have captured all of the commonly cited dimensions of access–affordability, acceptability, availability, and accessibility [75]. The indicators in this review may not sufficiently capture the additional complexities that people with disabilities may face in seeking and receiving health care of high quality. Our primary interest was receipt of health care, and using this approach we may have missed details about the quality and effectiveness of care received. Although we extracted data on barriers and quality as secondary outcomes, qualitative evidence may be able to provide a more in-depth analysis of these aspects of access. Barriers to access such as lack or cost of transport may play a more crucial role in access to health for people with disabilities than insurance coverage. Particularly as insurance may not cover all required services. The review was unable to examine in depth the influence that health financing, or health system performance has on access to health for people with disabilities. The outcomes were too varied to allow meaningful comparisons to be made. Further, the review found a trend for higher utilisation for people with disabilities, which is not unexpected given that people with disabilities tend to have greater health needs than people without disabilities. However, we have not captured information in this review about the availability of health services, from the health systems perspective—i.e., the types of services offered to people with disabilities and ability of the health workforce to meet population need. These factors are important to ensuring equitable access to health services for people with disabilities. The mixed results found in this study may underestimate the differences in access to health from an equity perspective. 

The searches were conducted in the English language and thus publications not in the English language may have been missed. Further, as 30% of publications were conducted in sub-Saharan Africa, our results may have a bias towards the conditions in these countries. 54% of studies in this review were judged to have a low risk of bias, with the remaining having high or moderate risk of bias. When interpreting the findings of this review, this must be taken in to consideration. Finally, as we focused on peer-reviewed empirical evidence, our review may have missed relevant information on access from grey literature sources. This review also has several strengths. The review followed PRISMA guidance, adopting a thorough approach to screening, data extraction and analysis of the results. 

## 5. Conclusions

This review summarises the available literature on access to general healthcare services for people with disabilities in LMIC. Although 50 studies were included in the review, the wide range of outcomes and methods for measuring disability made it difficult to draw strong conclusions. Developing common metrics for measuring disability and healthcare access, will improve the availability of high quality, comparable data. Providing good access to health for people with disabilities will ensure that their rights are met and help in achieving good health. This will also help in efforts towards achievement of UHC—by ensuring that healthcare services reach the whole population, so that they can experience better health, better productivity, and less poverty.

## Figures and Tables

**Table 1:** Quality assessment criteria and ratings.

**Assessment Criteria by Study Design**	
All study designs	
Study design, sampling method is appropriate to the study question Adequate sample size (>100 participants), or sample size calculations undertaken Response rate reported and acceptable (>70%) Disability/impairment measure is clearly defined and reliable Measure of access clearly defined and reliable Potential confounders taken into account in analysis Confidence intervals are presented	
Case control (additional criteria)	
Cases and controls are comparable Cases and controls are clearly defined	
Cohort (additional criteria)	
Groups being studied are comparable at baseline Losses to follow up are presented and acceptable	
**Overall Ratings**	
++	Low risk of bias: All or almost all of the above criteria were fulfilled, and those that were not fulfilled were thought unlikely to alter the conclusions of the study
+	Medium risk of bias: Some of the above criteria were fulfilled, and those not fulfilled were thought unlikely to alter the conclusions of the study
−	High risk of bias: Few or no criteria were fulfilled, and the conclusions of the study were thought likely or very likely to alter with their inclusion.

**Table 2:** Characteristics of included studies.

Variable	Category	No.	%
Region	Latin America/Caribbean	12	24
	East Asia/Pacific	6	12
	Sub-Saharan Africa	15	30
	Middle east	2	4
	South Asia	6	12
	Europe/Central Asia	1	2
	Various	8	16
Income level	Low	8	16
	Lower middle	17	34
	Upper middle	17	34
	Mixed	8	16
Location	Urban	15	30
	Rural	4	8
	Both	24	48
	Unclear	7	14
Decade of publication	1990	1	2
	2000	8	16
	2010	41	82
Study design	Cross-sectional	37	74
	Case-control study	13	26
Outcome measured	Utilisation	20	40
	Coverage	22	44
	Expenditure	8	16
	Insurance	5	10
	Adherence	3	6
	Barriers	8	16
	Quality	3	6
Age group	All ages (includes >5 years; >4 years)	9	18
	Adults only (>18 years)	17	34
	Older adults (>40 years; >50 years; >60 years)	11	22
	Children only (0–18 years)	10	20
	Unclear age/not presented	3	6
Disability domain	Visual impairment	11	22
	Hearing impairment	15	30
	Physical impairment	15	30
	Mental impairment	24	48
	Functional difficulties *	24	48
	Assistance with activities of daily living	3	6
	Other (communication, sensory, albinism)	3	6
	Multiple domains	19	38

* Typically includes difficulties with hearing, vision, walking, self-care, communicating, and remembering or concentrating.

**Table 3:** Comparison of utilisation of primary health or secondary services between people with and without disabilities.

Study	Country	Age Group of Participants	Disability Domain	Time Period (Months)				Outcome		Result	Summary
				12	6	1–3	NS				
Trani et al. (2010), Trani et al. (2012) [13,14]	Afghanistan	>4 years	Multiple: Physical impairment, sensory, mental impairment (mental illness/intellectual impairment)	✓	▪	▪	▪	Health centre utilisation		People with disabilities 82%; No disability 84% (*p* > 0.05)	NS
Fialho et al. (2014) [15]	Brazil	≥60 years	Activities of Daily Living	✓	▪	▪	▪	Number of appointments in past 12 months (0–1; 2–4; 5 or more); Home visits by doctor in past 12 months		ADL Number of appointments: PR (5 or more vs. 0–1) = 1.1 (1.0, 1.3) PR (2–4 vs. 0–1) = 1.1 (0.9, 1.1) Home visits: ADL PR = 8.5 (4.2, 17.3)	+
Danquah et al. (2015) [16]	Haiti	≥5 years	Functional difficulties	✓	▪	▪	▪	Number of visits to health centre (No visits (base); versus ≥3 visits)		Adults: OR = 2.1 (1.0, 4.3) Children: OR = 1.3 (0.5, 2.9)	+
Albanese et al. (2011) [17]	Mexico, Peru, Cuba, Dominican Republic, Puerto Rico, Venezuela, China, India, Nigeria	>65 years	Multiple: Mental impairment (Dementia, depression), physical impairments, mobility restriction	✓	▪	▪	▪	Community health service use		Pooled prevalence ratio (all countries): Depression 1.2 (1.1, 1.4); Dementia 0.9 (0.9, 0.9); Physical impairment 1.4 (1.3, 1.5); Mobility restriction 1.0 (0.9, 1.1)	+
Marella et al. (2014) [18]	Bangladesh, Fiji	≥18 years	Functional difficulties	▪	✓	▪	▪	Access to health services		Bangladesh: People with disabilities 69%; No disability 66% (*p* < 0.001); Fiji: People with disabilities 82%; No disability 82% (*p* < 0.001)	−
Rodrigues et al. (2009) [19]	Brazil	>65 years	Functional difficulties	▪	✓	▪	▪	Medical visit at the primary health care unit	PR = 1.3 (1.2; 1.5)		+
Fujii et al. (2012) [20]	Brazil	>18 years	Mental impairment (depression)	▪	✓	▪	▪	Number of visits to traditional health care provider	Mean visits: People with disabilities Treated for depression 14.4 ± 20.6, People with disabilities not treated for depression 8.4 ± 10.5; No disability 3.3 ± 5.6 (*p* < 0.05)		+
Freire et al. (2009) [21]	Brazil	>15 years	Hearing impairment	▪	▪	✓	▪	Medical consultation	PR = 1.3 (1.1, 1.5); *p* = 0.007		+
Liu et al. (2009) [22]	China	≥65 years	Multiple: Mental impairment (Dementia), limiting physical illness (hearing impairment, physical impairment (limb or arthritis), and/or visual impairment (eye problem))	▪	▪	✓	▪	Use of community services	Dementia:urban OR = 0.9 (0.7, 1.2); rural OR = 1.5 (0.8, 3.1); Number of limiting physical illness: 1–2 vs. none: urban OR = 2.3 (1.8, 2.9), rural OR = 3.8 (2.1, 6.9) 2 or more vs none: urban OR = 3.7 (2.9, 4.8); rural OR = 8.3 (4.1, 17.0)		+
Twomey et al. (2015) [23]	China, Cuba, Dominican Republic, India, Mexico, Nigeria, Peru, Puerto Rico, Venezuela	≥65 years	Multiple: Mental impairment (Dementia, depression), functioning difficulties (concentrating/remembering, self-care, physical, communication, participation)	▪	▪	✓	▪	Previous health service utilisation	Depression severity PR=1.0 (1.01, 1.03); Functioning difficulties: PR = 1.01 (1.00, 1.03)		+
Andrade et al. (2002) [24]	Brazil	>18 years	Mental impairment (mental disorders)	▪	▪	✓	▪	General health service utilisation in the past month	Any psychiatric disorder 38% (SE = 2.8); No psychiatric disorder 24% (SE = 1.6)		+
Groce et al. (2006) [25]	Swaziland	≥18 years	Hearing impairment	▪	▪	▪	✓	Clinic attendance		People with disabilities 69%; No disability 87% (*p* < 0.05)	-
Moodley et al. (2015) [26]	South Africa	Adults	Functional difficulties	▪	▪	▪	✓	Length of time since last consultation: last 30 days; 1–5 months ago; 6–12 months ago; >1 and <2 years ago; 2–4 years ago; 5–10 years ago; >10 years ago; never		People with disabilities 44%; 15%; 8%; 16%; 5%; 2%; 2%; 10%No disability 20%; 14%; 8%; 29%; 10%; 4%; 2%; 13%*p* < 0.001; *p* = 0.17; *p* = 1.00; *p* < 0.001; *p* < 0.001; *p* < 0.001; *p* = 1.00; *p* < 0.001	+

+ higher utilization among people with disabilities; − lower; NS: no difference; ADL: activities of daily living; PR: prevalence ratio; OR: odds ratio; ✓ yes; ▪ no.

**Table 4:** Comparison of utilisation of hospital services between people with and without disabilities.

Study	Country	Age Group of Participants	Disability Domain	Time Period (Months)					Summary of Results	Hospitalisation	Outpatient Visit
				12	6	3	1	NS			
Trani et al. (2010, 2012) [14,29]	Afghanistan	>4 years	Multiple: Physical impairment, sensory, mental impairment (mental illness/intellectual impairment)	✓	▪	▪	▪	▪	Hospital admission (12 months): People with disabilities 80%; No disability 90%; *p* < 0.001	−	
Palmer et al. (2011, 2012) [30,31]	Vietnam	>5 years	Multiple: Physical impairment, hearing impairment, speaking, visual impairment, mental impairment (intellectual impairment, mental illness)	✓	▪	▪	✓	▪	Inpatient (past 12 months) OR = 1.7 (*p* ≤ 0.01) Outpatient (past month) OR = 1.1 (*p* = NS)	+	NS
Palmer et al. (2014) [32]	Vietnam	>5 years	Functional difficulties	✓	▪	▪	✓	▪	Inpatient visit in last 12 months mean 0.19 (SE = 0.12) Significantly higher than other groups studied (formal employee, person living in poverty, self-employed) Outpatient visit in the past month: mean 0.32 (SE = 0.015). Higher than other groups, but statistical test not reported.	+	NS
Murthy et al. (2014) [33]	India	>18 years	Multiple: Physical impairments, visual impairment, hearing impairment, mental impairment (intellectual impairment)	✓	▪	▪	▪	▪	Need to visit hospital (past year) OR 1.6 (0.9, 2.5), *p* = 0.05	+	
Castro et al. (2013) [28]	Brazil	>11 years	Multiple: Visual impairment, hearing impairment, physical impairment	✓	▪	▪	▪	▪	PR for hospitalisation No disability PR: 1.00 (base) Visual: PR: 0.9 (0.45, 1.6); NS Hearing: PR: 1.6 (0.9, 2.9); NS Physical: PR:3.8 (2.0, 7.1) Multiple: PR:3.3 (1.6, 6.6)	+	
Fialho et al. (2014) [15]	Brazil	≥60 years	Participation	✓	▪	▪	▪	▪	PR for hospitalization = 1.6 (1.2, 2.3)	+	
Freire et al. (2009) [21]	Brazil	>15 years	Hearing impairment	✓	▪	▪	▪	▪	PR for hospitalization = 2.1 (1.4, 3.1)	+	
Blay et al. (2008) [27]	Brazil	>60 years	Multiple: Physical impairment, mental impairment (mental health condition)	✓	✓	▪	▪	▪	Hospitalisations (12 months) Rheumatism OR = 0.9 (0.8, 1.1) Psychiatric morbidity OR = 1.4 (1.1, 1.9) Outpatient visit (6 months) Rheumatism OR = 1.1 (0.9, 1.3) Psychiatric morbidity OR = 1.1 (0.9, 1.2)	+	NS
Fujii et al. (2012) [20]	Brazil	>18 years	Mental impairment (mental health)	▪	✓	▪	▪	▪	Hospitalisations (6 months): People with disabilities treated for depression 24%; People with disabilities not treated for depression: 17%; Control 8%; *p* < 0.05	+	
Devendra et al. (2013) [34]	Malawi	2–9 years	Functional difficulties	▪	▪	▪	▪	✓	Overnight hospital admission OR = 2.7 (1.2, 6.2)	+	
Twomey et al. (2015) [23]	Various	>65 years	Functional difficulties	▪	▪	✓	▪	▪	Hospital admission (past 3 months) Depression severity PR = 1.1 (0.9, 1.3) Functioning: PR = 1.1 (1.02, 1.3)	+	
World report on disability (2011) [1]	Various (50 LMIC)	18+ years	Functional difficulties	✓	▪	▪	▪	▪	Overall, people with disabilities sought more inpatient and outpatient care in the last 5 years compared to people without disabilities, and this difference was evidence across both genders and all age groups, except in people aged 60+	+	+

+ higher utilization among people with disabilities; − lower; NS: no difference; LMIC: low and middle-income countries; PR: prevalence ratio; OR: odds ratio; ✓ yes; ▪ no.

**Table 5:** Summary of studies measuring coverage.

Study Author, Year	Country		Age Group of Participants	Disability Group	Outcome	People with Disabilities	People without Disabilities		Comparison	Summary
**Care seeking when ill**										
Kuper et al. (2014) [4]	30 LMIC		Children	Physical, mental, vision, communication, hearing	Serious illness in the last 12 months and if sought treatment	>97%	>97%		Not reported	NS
Kuper et al. (2015) [47]	Kenya		Children	Physical, epilepsy, visual, hearing, intellectual, functional difficulties	Took action when sick	83%	84%		OR = 1.2 (0.6, 2.2)	NS
Kuper et al. (2016) [48]	Tanzania		All ages	Functioning (WG)	Proportion seeking care when ill	94%	96%		Not reported	NS
Mactaggart et al. (2015) [49]	India and Cameroon		All ages	Functioning (WG)	Sought care if serious health problem				India a OR = 0.9 (0.3, 3.1); Cameroon a OR = 1.8 (0.7, 4.3)	NS
Wandera et al. (2015) [35]	Uganda		Older adults	Functioning (WG)	Access to healthcare in the last 30 days when ill	Overall: 70% Within domains: Communicating: 49% Seeing: 67% Hearing: 67% Walking: 63% Remembering or concentrating: 55% Self-care: 55%	Overall: 80% Communicating 76% Seeing 78% Hearing 77% Walking 80% Remembering or concentrating 77% Self-care 77%		*p* < 0.001 *p* = 0.01 *p* = 0.03 *p* = 0.14 *p* < 0.001 *p* < 0.001 *p* = 0.001	−
Trani et al. (2015) [50]	India		Not specified	Mental health	Could you receive healthcare when sick?	4%	3%		*p* = 0.28	NS
Emerson et al. (2017) [36]	25 LMICs		Children	Intellectual disability	Help sought for respiratory infection Help sought for fever	53% 48%	65% 50%		OR = 0.69 (0.59, 0.80) OR = 0.95 (0.86, 1.05)	−
Eide et al. (2015) [51]	Sudan; Namibia; South Africa; Malawi		Not specified	Functioning (WG)	Probability of not receiving necessary health care	0.19	0.07		Statistical test not shown—trend for higher probability of not receiving care	−
Marella et al. (2016) [52]	Philippines		≥18 years	Multiple: Visual impairment, hearing impairment, communication, physical impairment, mental impairment (cognitive, appearance, psychological distress)	Met need for general health services				OR = 0.5 (0.3, 0.7)	−
**Dental**										
Al Habashneh et al. (2012) [37]		Jordan	Children 12–16 years	Intellectual disability (Down’s syndrome)	Dental visits (parent report) Visit dentist: Never, irregular, regular	32%, 58%, 10%	16%, 51%, 34%		*p* < 0.01	−
El Khatib et al. (2014) [38]		Egypt	Children	Behavioural impairment (Autism)	Dental visits in the past year; difficulty finding a dentist (parent report)	44%; 64%	67%; 25%		*p* = 0.002	−
Rahim et al. (2014) [40]		Malaysia	Children	Intellectual disability (Down’s syndrome)	Received any dental treatment	49%	53%		*p* > 0.01	NS
Oredugba et al. (2006) [39]		Nigeria	Children 5–19 years	Multiple: mental disabilities (e.g., Down’s syndrome, autism); physical (cerebral palsy)	Previous dental attendance	4%	4%		*p* > 0.05	NS
**Maternal**										
Bernabe-Ortiz et al. (2016) [53]		Peru	All ages	Functioning (WG)	Accessing prenatal care for pregnancies in the past 5 years	100%	100%		*p* > 0.05	NS
Mactaggart et al. (2015) [49]		Cameroon	All ages	Functioning (WG)	Antenatal care/vaccines for women (yes; no)				OR = 0.6 (0.2, 2.1)	NS
Murthy et al. (2014) [54]		India	Adults	Physical, visual, hearing, intellectual	Baby delivered at hospital in the last 2 years	63%	72%		*p* = 0.15	NS
Trani et al. (2011) [29]		Sierra Leone	Adults	Functioning (bespoke tool)	Antenatal visit; Birth attended by professional; Delivery in hospital; Access to emergency care	94% 94% 87% 80%	84% 84% 76% 76%		*p* = 0.35 *p* = 0.07 *p* = 0.18 *p* = 0.82	NS
**Vaccination**										
Kuper et al. (2015) [47]		Kenya	Children	Physical, epilepsy, visual, hearing, intellectual, functioning	Child received vaccinations	97%	98%		OR = 1.3 (0.5, 3.5)	NS
Bernabe-Ortiz et al. (2016) [53]		Peru	All ages	Functioning (WG)	Vaccination of children born in the past 5 years	100%	100%		*p* > 0.05	NS
Sato et al. (2015) [41]		Brazil	Older adults	Mental impairment (depression); physical impairment (“bedridden”)	Influenza vaccination coverage (self-report): Depression; “bedridden”				PR = 0.9 (0.9, 1.0) PR = 1.0 (0.8, 1.3)	NS
Mactaggart et al. (2015) [49]		India	All ages	Functioning (WG)	Child vaccinated (yes; no)				OR = 1.8 (0.3, 11.9)	NS
Trani et al. (2011) [29]		Sierra Leone	Adults	Functioning (bespoke tool)	Not immunised	25%	11%		*p* = 0.003	−
**HIV**										
Abimanyi-Ochom et al. (2017) [42]	Uganda		Adults	Functioning (WG)	Month since last test			*p* < 0.05 for all comparisons		+
Bisol et al. (2008) [43]	Brazil		Children	Hearing impairment	Ever been tested	21%	8%	*p* = 0.08		NS
De Beaudrap et al. (2017) [55]	Cameroon		Adults	Functioning (WG)	Ever been tested	71%	77%	OR = 0.8 (0.6, 1.0)		NS
Trani et al. (2011) [29]	Sierra Leone		Adults	Functioning (bespoke tool)	Ever been tested	16%	20%	*p* < 0.001		−
**Other outcomes**										
Freire et al. (2009) [21]	Brazil		Adults	Hearing impairment	(1) Average time elapsed since the last Pap smear (2) Time since last prostate exam	(1) 24.3 (SD = 32.9) (2) 30.6 (SD = 28.6)	(1) 7.2 (SD 13.8) (2) 30.5 (SD = 24.1)	(1) *p* < 0.001 (2) *p* = 0.98 (NS)		−
Gottlieb et al. (2009) [44]	18 LMIC		Children	Functioning (Ten Questions)	Receipt of vitamin A supplements (ever received yes or no) (parent report)	28%	77%	No significance test reported		−

+ higher utilization among people with disabilities; − lower; NS no difference; PR prevalence ratio; OR odds ratio; LMIC: low and middle income countries; WG: Washington group questions

**Table 6:** Results of studies measuring adherence.

Study Author, Year	Country	Age Range	Disability Domain	Relevant Measures of Access	Measure Among People with Disabilities	Measure Among Controls	Measure of association	Summary
Hannass-Hancock et al. (2015) [45]	South Africa	18–88 years	Functional difficulties	Non-adherence to HIV treatment	-	-	Relative risk ratios (RR) Global limitation 1.1 (1.05–1.14) Mobility 1.3 (1.2, 1.5) Life activity 0.7 (0.4, 1.2); NS Cognition 1.1 (0.8, 1.5); NS Participation 1.2 (0.9, 1.5); NS Self-care 0.7 (0.3, 1.4); NS Activity limitations OR = 1.1 (1.1, 1.2)	−
Mollaoglu et al. (2015) [56]	Turkey	>18 years	Functional difficulties	Diet non adherence (mean (SD)) Fluid non adherence (mean (SD))	Severe level 3.22 (0.66) Severe level 3.88 (1.05)	2.46 (0.75) 3.28 (0.45)	*p* < 0.001 (comparing scores between no, mild, moderate, severe disability) OR = 3.6 (2.1, 6.1) (comparing high to low level of disability) *p* < 0.001 (comparing scores between no, mild, moderate, severe disability) OR = 2.9 (1.0, 1.2) (comparing high to low level of disability)	−
Tavares et al. (2013) [46]	Brazil	>60 years	Multiple: Participation Mental impairment (cognitive deficit, depression)	% low adherence	IADL: 33%; Depression: 31%; Cognitive: 27%	No IADL 26%; No depression 28%; No cognitive deficit 29%	PR = 1.3 (1.1, 1.5) *p* = 0.009 PR = 1.1 (0.9, 1.4) *p* = 0.49 PR = 0.9 (0.7, 1.2) *p* = 0.67	−

+ higher utilization among people with disabilities; − lower; NS no difference; PR prevalence ratio; OR odds ratio.

**Table 7:** Results of studies measuring insurance and expenditure.

Study Author, Year	Country	Age Range	Disability Domain	Relevant Measures of Access	Measure among People with Disabilities	Measure among Controls	Measure of Association	Summary
**Insurance**								
Alhajj et al. (2010) [60]	China	15–84 years	Multiple: Hearing impairment, visual impairment, physical impairment, mental impairment	Medical payment method: Out of pocket Government insurance Commercial insurance	80% 20% 0.7%	82% 20% 0.6%	*p* > 0.05	NS
Bernabe-Ortiz et al. (2016) [53]	Peru	≥5 years	Functional difficulties	Enrolled in insurance scheme	83%;	81%	OR = 0.9 (0.5, 1.6)	NS
Freire et al. (2009) [21]	Brazil	>15 years	Hearing impairment	Enrolled in health plan			PR = 1.1, 95%CI 1.0–1.3; *p* = 0.11	NS
Moodley et al. (2015) [26]	South Africa	“adults”	Functional difficulties	Medical aid receipt	10%	18%	*p* < 0.001	−
Palmer et al. (2011) and Palmer (2012) [30,31]	Vietnam	>5 years	Multiple: Physical impairment, hearing impairment, speaking, visual impairment, mental impairment	Insurance card holder (mean)	0.19 (SE = 0.007)	0.18 (SE = 0.003)	*p* > 0.05	NS
**Expenditure**								
Brinda et al. (2012) [57]	India	>60 years	Multiple: Functional difficulties; mental impairment (Alzheimers, Dementia, Mental Health), hearing impairment, visual impairment	Total out of pocket health expenditure; catastrophic health expenditure			Correlates of out of pocket health expenditure Visual impairment *p* = 0.82 Hearing impairment *p* = 0.14 Dementia *p* < 0.001 Major depression *p* < 0.001 WHODAS II *p* < 0.001 Correlates of catastrophic health expenditure Visual impairment; *p* = 0.78 Hearing impairment; *p* = 0.66 Dementia; *p* = 0.01	+
Brinda et al. (2014) [58]	Tanzania	≥18 years	Multiple: Visual impairment, hearing impairment, functional difficulties, mental impairment	Total out of pocket health expenditure; catastrophic health expenditure			Out of pocket health expenditure (18–59 years) Blindness/visual defect NS Hearing defect: *p* = 0.02 Limb defect: NS Psychiatric morbidity NS Functional disability: *p* < 0.001 Out of pocket health expenditure (>60 years) Blindness/visual defect: *p* = 0.01 Hearing defect: NS Limb defect: NS Psychiatric morbidity: NS Functional disability: *p* = 0.01 Catastrophic expenses functional disability: 1.19 (0.93, 1.51); NS	+
Brinda et al. (2015) [59]	India	≥65 years	Functional difficulties	Out of pocket health expenditure and catastrophic health expenditure			Disability was positively correlated with out-of-pocket health expenditure (*p* < 0.001) Catastrophic health expenditure was associated with depression: OR = 3.5 (1.5, 7.5); *p* = 0.004	+
Palmer et al. (2014) [32]	Vietnam	>5 years	Functional difficulties	Inpatient expenditure (past month) Outpatient expenditure (past month) Catastrophic health expenditure: 10% threshold; 20% threshold; 40% threshold	Inpatient: 401 (57) Outpatient: 51 (8) 10%: 50% 20%: 30% 40%: 12%	Inpatient: 35–235 Outpatient: 6–39 10%: 20–40% 20%: 10–20% 40%: 3–10%	Inpatient and outpatient expenditures: NS Outpatient visit in the past month: NS Effect of insurance on catastrophic health expenditure: NS	NS
Palmer et al. (2011) and Palmer (2012) [30,31]	Vietnam	>5 years	Multiple: Physical, hearing, speaking, visual, mental impairment	Expenditure ratio: Inpatient; outpatient			Expenditure ratio: Inpatient 1.7 (0.15) *p* ≤ 0.01 Outpatient 0.9 (0.07) *p* = NS	+
Trani et al. (2011) [29]	Sierra Leone	≥18 years	Multiple: Physical impairment, sensory disabilities, mental impairment	Health expenditure: % total average yearly HH income spent on health	severe 4%	3%	Not measured	NS
World report on disability (2011) [1]	Various (50 LMIC)	≥18 years	Functioning	Catastrophic health expenditure	M 31.2%; F 33%; 18–49 years: 33%; 50–59 years: 33%; 60+ years: 30%	M 20%; F 20%; 18–49 years: 20%; 50–59 years: 18%; 60+ years: 21%	For all comparisons, catastrophic health expenditure was higher among people with disabilities *p* < 0.05	+
Trani et al. (2010) and Trani et al. (2012) [13,14]	Afghanistan	>4 years	Functioning	Medical expenses (Afghanis) amongst those with severe difficulties	None: 75% 1–499: 15% 500–1999: 7% 2000–105,000: 3%	None: 76% 1–499: 15% 500–1999: 7% 2000–105,000: 8%	*p* > 0.05	NS

+ higher utilization among people with disabilities; − lower; NS no difference; PR prevalence ratio; OR odds ratio; SE standard error.

**Table 8:** Barriers to accessing health services reported in included studies.

Barrier	Al Habashneh et al. (2011) [37]	Badu et al. (2016) [61]	Danquah et al. (2015) [16]	Eide et al. (2015) [51]	Marella et al. (2016) [52]	Murthy et al. (2014) [33]	Rahim et al. (2014) [40]	World report on Disability (2011) [1]
**Geographic accessibility**								
Transport difficulties	✓		✓	✓		✓		✓
Location of services		✓						
**Affordability**								
Financial	✓		✓	✓	✓			✓
No accommodation at health facility				✓				
**Acceptability**								
Lack of perceived need				✓				✓
Other commitments				✓				✓
Lack of awareness or information	✓				✓			✓
Did not know where to go				✓		✓		
Fear of service	✓							
Fear of journey	✓			✓				
Faith/belief				✓				
Discrimination or lack of awareness amongst health workers		✓		✓		✓		✓
Previous bad experience				✓				
Communication with health providers				✓				
Standard of facility				✓				
Physical access to facility				✓		✓		
**Availability**								
Services not available				✓				
Lack of equipment				✓		✓		✓
Tried but denied				✓				✓
Health care providers skills inadequate								✓
Difficulty finding doctor							✓	

✓ yes.

## Supplementary Materials

The following are available online at http://www.mdpi.com/1660-4601/15/9/1879/s1, File S1: EMBASE search strategy, File S2: Data Extraction, File S3: Quality review results.
