# Supplementary material for: A Systematic Review of Access to General Healthcare Services for People with Disabilities in Low and Middle Income Countries
Source: Int J Environ Res Public Health. 2018 Aug 30;15(9):1879. doi: 10.3390/ijerph15091879 (PMC6164773; doi:10.3390/ijerph15091879)
Supplement: Supplementary file 1 [file ijerph-15-01879-s001.zip › Suppl. File 1.pdf]

EMBASE search strategy.

#	Searches	Results	Annotations
1	Developing.mp. [mp=title, abstract, heading word, drug trade name, original title, device manufacturer, drug manufacturer, device trade name, keyword, floating subheading word]	598110	
2	low-income.mp. [mp=title, abstract, heading word, drug trade name, original title, device manufacturer, drug manufacturer, device trade name, keyword, floating subheading word]	30476	
3	low income.mp. [mp=title, abstract, heading word, drug trade name, original title, device manufacturer, drug manufacturer, device trade name, keyword, floating subheading word]	30476	
4	middle-income.mp. [mp=title, abstract, heading word, drug trade name, original title, device manufacturer, drug manufacturer, device trade name, keyword, floating subheading word]	12451	
5	middle income.mp. [mp=title, abstract, heading word, drug trade name, original title, device manufacturer, drug manufacturer, device trade name, keyword, floating subheading word]	12451	
6	(low and middle income).mp. [mp=title, abstract, heading word, drug trade name, original title, device manufacturer, drug manufacturer, device trade name, keyword, floating subheading word]	10722	
7	less-developed.mp. [mp=title, abstract, heading word, drug trade name, original title, device manufacturer, drug manufacturer, device trade name, keyword, floating subheading word]	3357	
8	(low- and middle-income).mp. [mp=title, abstract, heading word, drug trade name, original title, device manufacturer, drug manufacturer, device trade name, keyword, floating subheading word]	10722	
9	less developed.mp. [mp=title, abstract, heading word, drug trade name, original title, device manufacturer, drug manufacturer, device trade name, keyword, floating subheading word]	3357	
10	under developed.mp. [mp=title, abstract, heading word, drug trade name, original title, device manufacturer, drug manufacturer, device trade name, keyword, floating subheading word]	685	
11	underdeveloped.mp. [mp=title, abstract, heading word, drug trade name, original title, device manufacturer, drug manufacturer, device trade name, keyword, floating subheading word]	5363	

12	third-world.mp. [mp=title, abstract, heading word, drug trade name, original title, device manufacturer, drug manufacturer, device trade name, keyword, floating subheading word]	3405	
13	third world.mp. [mp=title, abstract, heading word, drug trade name, original title, device manufacturer, drug manufacturer, device trade name, keyword, floating subheading word]	3405	
14	1 or 2 or 3 or 4 or 5 or 6 or 7 or 8 or 9 or 10 or 11 or 12 or 13	640660	
15	((Developing or low-income or low income or middle-income or middle income or (low and middle income) or less-developed or (low- and middle-income) or less developed or under developed or underdeveloped or third-world or third world) adj5 (countr* or nation* or world or econom*)).mp.	146531	
16	exp Developing Countries/	87090	
17	(LIC or LICs or MIC or MICs or LMIC or LMICs or LAMIC or LAMICs or LAMI countr*).mp. [mp=title, abstract, heading word, drug trade name, original title, device manufacturer, drug manufacturer, device trade name, keyword, floating subheading word]	56705	
18	(Transitional countr* or Transitional econom* or Transition countr* or Transition econom*).mp. [mp=title, abstract, heading word, drug trade name, original title, device manufacturer, drug manufacturer, device trade name, keyword, floating subheading word]	437	
19	(Africa or Asia or Caribbean or West Indies or Latin America or Central America or South America).mp. [mp=title, abstract, heading word, drug trade name, original title, device manufacturer, drug manufacturer, device trade name, keyword, floating subheading word]	300779	
20	exp Africa South of the Sahara/ or exp Africa/ or exp Asia, Central/ or exp Asia, South East/ or exp Asia, Western/ or exp Latin America/ or exp Caribbean/ or exp Central America/ or exp South America/	1272648	
21	(Afghanistan or Albania or Algeria or American Samoa or Angola or Antigua or Barbuda or Argentina or Armenia or Azerbaijan or Bangladesh or Belarus or Byelarus or Byelorussia or Belorussia or Belize or Benin or Bhutan or Bolivia or Bosnia or Herzegovina or Hercegovina or Bosnia-Herzegovina or Bosnia-Hercegovina or Botswana or Brazil or Brasil or Bulgaria or Burkina or Upper Volta or Burundi or Urundi or Cambodia or Republic of Kampuchea or Cameroon or Cameroons or Cape Verde or Central African Republic or Chad or Chile or China or Colombia or Comoros or Comoro Islands or Comores or Congo or DRC or Zaire or Costa Rica or Cote d'Ivoire or Ivory Coast or Cuba or Djibouti or Obock or French Somaliland or Dominica or Dominican Republic or	1608277	

	Ecuador or Egypt or United Arab Republic or El Salvador or Eritrea or Ethiopia or Fiji or Gabon or Gabonese Republic or Gambia or Georgia or Ghana or Gold Coast or Grenada or Guatemala or Guinea or Guinea-Bissau or Guiana or Guyana or Haiti or Honduras or India or Indonesia or Iran or Iraq or Jamaica or Jordan or Kazakhstan or Kenya or Kiribati or Republic of Korea or North Korea or DPRK or Kosovo or Kyrgyzstan or Kirghizstan or Kirgizstan or Kirghizia or Kirgizia or Kyrgyz or Kirghiz or Kyrgyz Republic or Lao or Laos or Latvia or Lebanon or Lesotho or Basutoland or Liberia or Libya or Lithuania or Macedonia or Madagascar or Malagasy Republic or Malawi or Nyasaland or Malaysia or Malaya or Malay or Maldives or Mali or Marshall Islands or Mauritania or Mauritius or Mayotte or Mexico or Micronesia or Moldova or Moldovia or Mongolia or Montenegro or Morocco or Mozambique or Myanmar or Burma or Namibia or Nepal or Nicaragua or Niger or Nigeria or Pakistan or Palau or Palestine or Panama or Papua New Guinea or Paraguay or Peru or Philippines or Romania or Rumania or Roumania or Russia or Russian Federation or USSR or Soviet Union or Union of Soviet Socialist Republics or Rwanda or Ruanda-Urundi or Samoa or Samoan Islands or Sao Tome or Principe or Senegal or Serbia or Montenegro or Yugoslavia or Seychelles or Sierra Leone or Solomon Islands or Somalia or South Africa or Sri Lanka or Ceylon or Saint Kitts or St Kitts or Saint Christopher Island or Nevis or Saint Lucia or St Lucia or Saint Vincent or St Vincent or Grenadines or Sudan or Suriname or Surinam or Swaziland or Syria or Syrian Arab Republic or Tajikistan or Tadzhikistan or Tadjikistan or Tanzania or Thailand or Timor-Leste or East Timor or Togo or Togolese Republic or Tonga or Tunisia or Turkey or Turkmenistan or Turkmenia or Tuvalu or Uganda or Ukraine or Uruguay or Uzbekistan or Vanuatu or New Hebrides or Venezuela or Vietnam or Viet Nam or West Bank or Gaza or Yemen or Zambia or Zimbabwe or Rhodesia).mp. [mp=title, abstract, heading word, drug trade name, original title, device manufacturer, drug manufacturer, device trade name, keyword, floating subheading word]		
22	15 or 16 or 17 or 18 or 19 or 20 or 21	2149386	
23	((person* with disabilit* or people with disabilit* or (disable* or Disabilit* or Handicap*)) adj5 (person* or people)).mp. [mp=title, abstract, heading word, drug trade name, original title, device manufacturer, drug manufacturer, device trade name, keyword, floating subheading word]	51530	
24	(Physical* adj5 (impair* or deficienc* or disable* or disabili* or handicap*)).mp. [mp=title, abstract, heading word, drug trade name, original title, device	38130	

	manufacturer, drug manufacturer, device trade name, keyword, floating subheading word]		
25	(Cerebral pals* or Spina bifida or Muscular dystroph* or Arthriti* or Osteogenesis imperfecta or Musculoskeletal abnormalit* or Musculo-skeletal abnormalit* or Muscular abnormalit* or Skeletal abnormalit* or Limb abnormalit* or Amputation* or Clubfoot or Poliomyeliti* or Paraplegi* or Paralys* or Paralyz* or Hemiplegi*).mp. [mp=title, abstract, heading word, drug trade name, original title, device manufacturer, drug manufacturer, device trade name, keyword, floating subheading word]	687454	
26	((Hearing or Acoustic or Ear$3) adj5 (loss* or impair* or deficienc* or disable* or disabili* or handicap*)).mp. [mp=title, abstract, heading word, drug trade name, original title, device manufacturer, drug manufacturer, device trade name, keyword, floating subheading word]	137842	
27	((Visual* or Vision or Eye$3) adj5 (loss* or impair* or deficienc* or disable* or disabili* or handicap*)).mp. [mp=title, abstract, heading word, drug trade name, original title, device manufacturer, drug manufacturer, device trade name, keyword, floating subheading word]	86190	
28	(Deaf* or Blind*).mp. [mp=title, abstract, heading word, drug trade name, original title, device manufacturer, drug manufacturer, device trade name, keyword, floating subheading word]	480529	
29	exp Hearing impairment/ or exp vision disorders/ or exp Deafness/ or exp Blindness/	294763	
30	(Schizophreni* or Psychosis or Psychoses or Psychotic Disorder* or Schizoaffective Disorder* or Schizophreniform Disorder* or Dementia* or Alzheimer*).mp. [mp=title, abstract, heading word, drug trade name, original title, device manufacturer, drug manufacturer, device trade name, keyword, floating subheading word]	540291	
31	exp Schizophrenia/	173735	
32	exp Dementia/ or exp Alzheimer disease/	294153	
33	31 or 32	459369	
34	((Intellectual* or Mental* or Psychological* or Developmental) adj5 (impair* or retard* or deficienc* or disable* or disabili* or handicap* or ill?6)).mp. [mp=title, abstract, heading word, drug trade name, original title, device manufacturer, drug manufacturer, device trade name, keyword, floating subheading word]	130687	
35	((communication or language or speech or learning) adj5 disorder*).mp. [mp=title, abstract, heading word, drug trade name, original title, device	67030	

	manufacturer, drug manufacturer, device trade name, keyword, floating subheading word]		
36	exp Disabled Children/	8489	
37	exp Learning Disorders/	32681	
38	exp Mentally Disabled Persons/	184	
39	36 or 37 or 38	41077	
40	23 or 24 or 25 or 26 or 27 or 28 or 29 or 30 or 33 or 34 or 35 or 39	2167263	
41	40 not (disability adjusted life year* or DALY).mp. [mp=title, abstract, heading word, drug trade name, original title, device manufacturer, drug manufacturer, device trade name, keyword, floating subheading word]	2166778	
42	41 not (double blind* or single blind* or blinded study).mp. [mp=title, abstract, heading word, drug trade name, original title, device manufacturer, drug manufacturer, device trade name, keyword, floating subheading word]	1909397	
43	exp Insurance, Health/	221903	
44	exp Palliative Care/	89105	
45	exp Preventive Health Services/	25024	
46	exp Child Health Services/	85873	
47	exp Maternal Health Services/	307	
48	exp Primary Health Care/	135154	
49	exp Health Services/	4520626	
50	exp Health Promotion/	82697	
51	exp Immunization/	272930	
52	exp Immunization Programs/	25024	
53	exp Rehabilitation/	330424	
54	exp Family Planning Services/	36282	
55	exp Reproductive Health Services/	4520626	
56	exp Mass Screening/	196915	
57	exp Prenatal Care/	132573	
58	exp Breast Feeding/	43466	
59	exp Smoking Cessation/	47753	
60	exp Anti-HIV Agents/	137207	
61	exp Insecticide-Treated Bednets/	303	
62	exp Fluid Therapy/	80769	
63	exp Hand Disinfection/	86	

64	(access* or equal* or inequal* or barrier*).mp. [mp=title, abstract, heading word, drug trade name, original title, device manufacturer, drug manufacturer, device trade name, keyword, floating subheading word]	1196730	
65	exp Papillomavirus Vaccines/	9990	
66	exp Hepatitis B Vaccines/	17524	
67	47 or 54 or 55 or 57 or 58 or 59 or 60 or 61 or 62 or 63 or 65 or 66	4831428	
68	43 or 44 or 45 or 46 or 48 or 49 or 50 or 51 or 52 or 53	4938817	
69	64 and 68	310147	
70	22 and 42 and 69	3229	
71	67 or 68	5228079	
72	64 and 71	324011	
73	22 and 42 and 72	3321	
74	exp Tuberculosis/	234046	
75	(prevent* or treat* or diagn*).mp. [mp=title, abstract, heading word, drug trade name, original title, device manufacturer, drug manufacturer, device trade name, keyword, floating subheading word]	12400328	
76	74 and 75	150547	
77	exp Antihypertensive Agents/	653359	
78	67 or 76 or 77	5482294	
79	68 or 78	5869969	
80	64 and 79	341918	
81	22 and 42 and 80	3428	
82	from 73 keep 1-1039	1039	
83	from 81 keep 1-1065	1065	
84	56 or 68	5025074	
85	84 or health*.mp. [mp=title, abstract, heading word, drug trade name, original title, device manufacturer, drug manufacturer, device trade name, keyword, floating subheading word]	7384455	
86	78 or 85	8209053	
87	64 and 86	464932	
88	22 and 42 and 87	4691	
